# Development of the Wheat Practical Haplotype Graph database as a resource for genotyping data storage and genotype imputation

**DOI:** 10.1093/g3journal/jkab390

**Published:** 2021-11-09

**Authors:** Katherine W Jordan, Peter J Bradbury, Zachary R Miller, Moses Nyine, Fei He, Max Fraser, Jim Anderson, Esten Mason, Andrew Katz, Stephen Pearce, Arron H Carter, Samuel Prather, Michael Pumphrey, Jianli Chen, Jason Cook, Shuyu Liu, Jackie C Rudd, Zhen Wang, Chenggen Chu, Amir M H Ibrahim, Jonathan Turkus, Eric Olson, Ragupathi Nagarajan, Brett Carver, Liuling Yan, Ellie Taagen, Mark Sorrells, Brian Ward, Jie Ren, Alina Akhunova, Guihua Bai, Robert Bowden, Jason Fiedler, Justin Faris, Jorge Dubcovsky, Mary Guttieri, Gina Brown-Guedira, Ed Buckler, Jean-Luc Jannink, Eduard D Akhunov

**Affiliations:** 1 Department of Plant Pathology, Kansas State University, Manhattan, KS 66506, USA; 2 USDA-ARS, Hard Winter Wheat Genetics Research Unit, Manhattan, KS 66502, USA; 3 USDA-ARS, Plant Soil and Nutrition Research Unit, Ithaca, NY 14853, USA; 4 Institute for Genomic Diversity, Cornell University, Ithaca, NY 14853, USA; 5 Department of Agronomy and Plant Genetics, University of Minnesota, St. Paul, MN 55108, USA; 6 Department of Soil and Crop Sciences, Colorado State University, Fort Collins, CO 80521, USA; 7 Department of Crop and Soil Sciences, Washington State University, Pullman, WA 99164, USA; 8 Department of Plant Sciences, University of Idaho, Aberdeen, ID 83210, USA; 9 Department of Plant Sciences and Plant Pathology, Montana State University, Bozeman, MT 59717, USA; 10 Department of Soil and Crop Sciences, Texas A&M AgriLife Research, Amarillo, TX 79106, USA; 11 Department of Plant, Soil and Microbial Sciences, Michigan State University, East Lansing, MI 48824, USA; 12 Department of Plant and Soil Sciences, Oklahoma State University, Stillwater, OK 74075, USA; 13 USDA-ARS, Plant Science Research Unit, Raleigh, NC 27695, USA; 14 Integrative Genomics Facility, Kansas State University, Manhattan, KS 66506, USA; 15 USDA-ARS, Cereal Crops Research Unit, Fargo, ND 58102, USA; 16 Department of Plant Sciences, University of California-Davis, Davis, CA 95616, USA

**Keywords:** wheat, genotype imputation, Practical Haplotype Graph, skim-seq, exome capture

## Abstract

To improve the efficiency of high-density genotype data storage and imputation in bread wheat (*Triticum aestivum* L.), we applied the Practical Haplotype Graph (PHG) tool. The Wheat PHG database was built using whole-exome capture sequencing data from a diverse set of 65 wheat accessions. Population haplotypes were inferred for the reference genome intervals defined by the boundaries of the high-quality gene models. Missing genotypes in the inference panels, composed of wheat cultivars or recombinant inbred lines genotyped by exome capture, genotyping-by-sequencing (GBS), or whole-genome skim-seq sequencing approaches, were imputed using the Wheat PHG database. Though imputation accuracy varied depending on the method of sequencing and coverage depth, we found 92% imputation accuracy with 0.01× sequence coverage, which was slightly lower than the accuracy obtained using the 0.5× sequence coverage (96.6%). Compared to Beagle, on average, PHG imputation was ∼3.5% (*P*-value < 2 × 10^−14^) more accurate, and showed 27% higher accuracy at imputing a rare haplotype introgressed from a wild relative into wheat. We found reduced accuracy of imputation with independent 2× GBS data (88.6%), which increases to 89.2% with the inclusion of parental haplotypes in the database. The accuracy reduction with GBS is likely associated with the small overlap between GBS markers and the exome capture dataset, which was used for constructing PHG. The highest imputation accuracy was obtained with exome capture for the wheat D genome, which also showed the highest levels of linkage disequilibrium and proportion of identity-by-descent regions among accessions in the PHG database. We demonstrate that genetic mapping based on genotypes imputed using PHG identifies SNPs with a broader range of effect sizes that together explain a higher proportion of genetic variance for heading date and meiotic crossover rate compared to previous studies.

## Introduction

For the last 10,000 years, intensive selection of bread wheat, *Triticum aestivum*, created varieties adapted to diverse environments and cultivation practices (Balfourier *et al.* 2019; He *et al.* 2019; Walkowiak *et al.* 2020). Recent advances in crop genomics and the availability of reference genomes have accelerated the adoption of sequence-based genotyping technologies for studying the genetics of agronomic traits (Nyine *et al.* 2019) and local adaptation (He *et al.* 2019; Juliana *et al.* 2019, 2020) and facilitated the introduction of genomics-assisted breeding strategies into wheat improvement pipelines (Poland and Rife 2012; Isidro *et al.* 2015). However, the limited genome coverage provided by these genotyping technologies does not support the exploration of the entire range of genetic effects conferred by all variants, limiting the utility of the developed genomic diversity and functional genomics resources for understanding genome-to-phenome connections. 

The large size (17 Gb) and complexity of the wheat genome present a substantial challenge for sequence-based analysis of genetic diversity. Alignment of short sequence reads to the wheat genome is complicated by high levels of sequence redundancy resulting from two rounds of recent whole genome duplication (IWGSC 2018), and the recent propagation of transposable elements comprising nearly 90% of the genome (Wicker *et al.* 2018). Therefore, the efforts of the wheat research community were focused primarily on sequencing complexity-reduced genomic libraries produced by either enzymatic digests or by targeted sequence capture. These efforts have resulted in a detailed description of the population-scale haplotypic diversity in the low-copy genomic regions in large sets of genetically and geographically diverse wheat lines and breeding populations (He *et al.* 2019; Juliana *et al.* 2019; Pont *et al.* 2019). While these resources have been useful for genotype imputation in populations genotyped using either SNP-based arrays or genotyping-by-sequencing (GBS) methods (Jordan *et al.* 2015; Shi *et al.* 2017; Juliana *et al.* 2019; Nyine *et al.* 2019), the relatively small number of shared markers between the reference and inference populations limits the number of imputed genotypes, thus diminishing the utility of genotype imputation in wheat genetic studies and breeding.

High-quality reference genomes and a reduction in the cost of sequencing presented opportunities for the characterization of genetic diversity by direct sequencing of either whole genomes or genomic regions targeted by sequence capture (Malmberg *et al.* 2018; He *et al.* 2019; Walkowiak *et al.* 2020). While these sequence-based genotyping approaches generate unbiased information about the genetic variants of various frequency classes and genomic locations, large-scale population sequencing of species with large genomes, including many important agricultural crops, remains costly. This issue has been addressed by combining low-coverage sequencing of whole genomes with the prediction of missing genotypes using imputation tools, thereby increasing the power of association mapping and facilitating the detection of causal variants (Davies *et al.* 2016; Das *et al.* 2018; Rubinacci *et al.* 2021).

Recently, a novel strategy referred to as Practical Haplotype Graph (PHG) was proposed to improve the efficiency of sequence-based genotyping data storage and imputing genotypes in low-coverage sequencing datasets (Jensen *et al.* 2020; Valdes Franco *et al.* 2020). The PHG is capable of storing sequencing data generated using diverse genotyping technologies as a graph of haplotypes of founder lines and is used for predicting missing genotypes in populations characterized by various sequence- or array-based genotyping strategies. By reducing the constraints associated with large-scale sequencing data storage, processing, and utilization, this tool is another step toward leveraging the existing community-generated genomic diversity resources in breeding and research applications. We used skim-seq, whole-exome capture, GBS, and array-based genotyping datasets generated by the USDA-NIFA WheatCAP to develop a Wheat PHG database and evaluate its performance for genotype imputation in wheat lines of different levels of relatedness and different depths of genome coverage.

## Materials and methods

The purpose of this paper is to assess the practicality and effectiveness of imputation using the Practical Haploytpe Graph (PHG) database tool in allohexaploid wheat with the complex genome. Our study combines five datasets that were created using different sequencing approaches. A summary table describing the datasets and their usage is provided in Supplementary Table S1.

### Datasets

#### WC65:

The primary dataset used in this study includes 65 wheat accessions and breeding lines that were subjected to whole exome capture as part of the WheatCAP, henceforth referred to as WC65. Many of these lines are used as parents in the US university/academia-associated wheat breeding programs, and information about these lines is found in Supplementary Table S2.

##### Sequencing library prep for WC65

DNA was extracted from the leaves of 2-week seedlings grown under greenhouse conditions. DNA was extracted using Qiagen DNeasy kit following the manufacturer’s protocol. DNA was quantified with Picogreen (Sage Scientific) and wheat exome capture was performed on each sample targeting the non-redundant low-copy portion of the genome. Briefly, wheat exome captures designed in collaboration with Nimblegen targeted 170 Mb of sequence covering about 80,000 transcripts (Krasileva *et al.* 2017). The barcoded genomic libraries were pooled at 12- or 96-plex levels, and sequenced on NextSeq (Kansas State University (KSU) Integrated Genomics Facility) and/or NovaSeq (Kansas University Medical Center) instrumentation using 2 × 150 bp read runs to produce sequence data providing about 30× coverage of the exome capture target space.

##### Data processing of WC65

The quality of sequence reads was assessed using NGSQC toolkit v.2.3.3 (Patel and Jain 2012). The sequence reads were aligned to the wheat reference genome RefSeq v.1.1 (IWGSC 2018) using HISAT2 (Kim *et al.* 2015) retaining only uniquely mapped reads. The resulting alignments were processed using the GATK pipeline (McKenna *et al.* 2010) to generate a genome variant call file (g.vcf format) for each accession. These g.vcf files were used to populate the PHG database (see below). The PHG pipeline exported a variant call file (.vcf format), containing 1,473,670 variable sites, which was subsequently used for diversity analyses, and to assess the accuracy of imputation using both the PHG and Beagle5.0 (see below).

##### Diversity analysis on WC65

Diversity statistics (π and Tajima’s D) were calculated using TASSEL v5.2.65 (Bradbury *et al.* 2007) in sliding windows of 2000 SNPs per window stepping 1000 SNPs at a time. The identity-by-descent (IBD) segments were identified using Beagle v.4.1 with the default parameters (Browning and Browning 2013), and considered to be significant at LOD ≥ 3.0. Overlap between the IBD segments was determined using the MultiIntersectBed tool of the Bedtools suite v.2.26.0 (Quinlan and Hall 2010). Linkage disequilibrium (LD) was determined using PLINK v.1.90b3.45 (Purcell *et al.* 2007) by calculating the squared correlation coefficient *r*^2^ for all possible pairwise combinations of SNP sites from the same chromosomes.

#### DS75

The second dataset used in our study includes another set of US breeding lines subjected to exome capture at KSU Integrated Genomics Facility. Information about these lines is found in Supplementary Table S2. This dataset was used to test the imputation efficiency and accuracy of the PHG database at reduced genome coverage depths.

##### Sequencing Library prep for DS75

DNA was extracted from leaf tissue as stated above for the WC65. The samples were subjected to whole exome capture and sequenced on the NovaSeq (Kansas University Medical Center) platform using 2 × 150 bp read runs, generating ∼30× depth of coverage.

##### Data processing of DS75

To assess the effect of genome coverage depth on imputation accuracy, we used *seqtk* (Li 2012) to generate three distinct down-sampled datasets from the 170 Mb wheat exome capture data to mimic 0.01× [5,667 paired-end (PE) reads per accession], 0.1× (56,667 PE per accession), and 0.5× (283,333 PE reads per accession) depth of coverage for the DS75 breeding lines (Supplementary Table S2). This set of DS75 breeding lines included four lines (Duster, Overley, NuPlains, and Zenda), which were also used to build the PHG database, and were part of the WC65 dataset. For each low-coverage level, fastq files of the DS75 accessions were run through the PHG imputation pipeline step (see PHG imputation below).

To impute using Beagle5.0 (Browning and Browning 2013) at low-coverage levels (0.1× and 0.01×), fastq files of the DS75 accessions were aligned to the wheat reference genome RefSeq v.1.1 (IWGSC 2018) using HISAT2 (Kim *et al.* 2015) retaining only uniquely mapped reads. The resulting alignments were processed using the GATK pipeline (McKenna *et al.* 2010) and combined to produce a vcf file at each coverage level, which were used as the target files for Beagle imputation. Imputation of the DS75 target panel was run using Beagle5.0 (Browning and Browning 2013) with a window size of 75 Mb and overlap size of 5 Mb, and the WC65 variant data was used as the reference panel. The imputed genotypes in the DS75 data generated using Beagle5.0 and PHG were compared at each coverage level.

##### Imputation accuracy of DS75

To test the accuracy of imputation in the low-coverage datasets from DS75, high coverage exome capture data generated for DS75 accessions were used to select a HQ-SNP dataset. The ∼30× exome capture sequenced reads were aligned to RefSeq v.1.1 (IWGSC 2018) and variants called using the approaches described above for the WC65 dataset. The raw GATK pipeline SNPs were filtered using *bcftools* (Danecek *et al.* 2021) retain variants with minor allele frequency ≥ 0.015 and missing data < 10%. Filtered GATK variants were combined with the 90K genotyping data (Wang *et al.* 2014), producing high-quality filtered variants (henceforth, HQ-SNPs) that were used for assessing the accuracy of the imputation for each accession.

The concordance of imputed genotypes was assessed in relation to the HQ-SNPs using a custom Perl script. The script compares the SNP positions and alleles between the imputed and HQ-SNP datasets for each accession, and divides the number of matching genotype calls by the total number of overlapped genotype calls. On average, the estimates of accuracy were based on nearly 550,000 genotype calls per accession for DS75. The imputation accuracy in DS75 between the Beagle v.5.0 and PHG imputation methods for 0.01× and 0.1× coverage levels was compared using a paired *t*-test. At each coverage level, PHG imputation was more accurate (0.01×: *t *=* *9.59, *P*-value = 1.9 × 10^−14^; 0.1×: *t *=* *19.06, *P*-value = 2.0 × 10^−16^) than Beagle imputation. Imputation accuracy comparisons between genomes and SNPs with different minor allele frequencies (MAFs) were performed using ANOVA from *car* and *lme4* R packages.

#### GBS70

A GBS sequencing dataset using MspI-PstI digested DNA of 70 wheat accessions were sequenced using GBS and whole exome capture, to check imputation accuracy on an independent GBS dataset (Supplementary Table S2). These lines were not included into the PHG database construction. An *in silico* digestion of wheat genome RefSeq v.1.0 detected nearly 3 million PstI recognition sites, of which 1.96 million are located within 250 bp of an MspI recognition site (Bernardo *et al*. 2020), and given GBS sequencing read lengths are 100 bp, we estimate the target size of GBS sequencing is 196 Mb. The majority (52 accessions) of these accessions were sequenced at 2.5× coverage, while 18 accessions were sequenced at a slightly lower coverage depth (∼1× target space), providing a chance to compare PHG imputation using GBS sequencing data providing different coverage depths of targeted sites.

##### Data processing of GBS70

Raw fastq files (1 × 100 bp) were quality filtered, separated by barcode, and barcodes trimmed from reads, as described (Jordan *et al.* 2018). Trimmed fastq files were processed using the PHG imputation pipeline (see *PHG imputation* below).

##### Imputation accuracy of GBS70

The accuracy of PHG imputation was assessed by calculating concordance between imputed genotypes and genotypes from the HQ-SNP dataset. On average, the estimates of accuracy were based on nearly 550,000 genotype calls per accession for GBS70.

#### NAMgbs

Previously generated GBS data (Jordan *et al.* 2018) based on MseI-PstI-digested DNA (Saintenac *et al.* 2013) from the wheat nested association mapping (NAM) population were used to test the imputation accuracy of the Wheat PHG. This dataset includes 2100 recombinant inbred lines (RILs) that represent a population of 28 families of 75 RILs each. The common parent, Berkut, and three other NAM parental lines, including Dharwar Dry, PBW343, and PI382150 (Supplementary Table S2), were used in the PHG construction.

##### Data processing of NAMgbs

Fastq files (1 × 100 bp) were processed as previously described (Jordan *et al.* 2018). On average, our dataset included 1.85 million reads per accession, corresponding to ∼1× coverage of the PstI-MseI sites in the reference wheat genome. The fastq files were processed using the PHG imputation pipeline (see below).

##### Imputation accuracy of NAMgbs

The concordance of imputed genotypes from the PHG pipeline was assessed by comparing with the previously reported, high-quality 90K iSelect genotyping data (Wang *et al.* 2014) generated for the NAM population, and high-quality SNPs identified in the NAM population. These high-quality SNPs were identified using the same procedures applied for the DS75 lines, except for including a post-GATK filtering step that retained only those SNPs that segregate among the NAM parents, and have MAF > 0.015 (henceforth, HQ-NAM SNPs). On average, the estimates of accuracy in the NAMgbs dataset were based on nearly 5000 genotype calls per accession. The comparisons of the imputation accuracy between families where both parents were used to construct the PHG database and families with only one parent represented in the PHG database were performed using ANOVA.

#### NAMskim

Genomic libraries of low-coverage whole-genome skim sequencing (Malmberg *et al.* 2018) were prepared for 24 samples (Supplementary Table S2) from one of the NAM families (Jordan *et al.* 2018) using Illumina DNA Prep Kit along with the Illumina’s Nextera CD adapters. Sequencing (2 × 150 bp) was performed on the Illumina NextSeq platform (Kansas State University, Integrated Genomics Facility) for an average of 6.1 million PE reads per accession, which represents ∼0.1× genome coverage.

##### Data processing of NAMskim

Demultiplexed fastq files were quality trimmed and used for PHG imputation (see *PHG imputation* below). The accuracy of PHG imputation was assessed by calculating the concordance of imputed genotypes and genotypes from the HQ-NAM dataset. On average, the estimates of accuracy were based on nearly 5000 genotype calls per accession. Paired *t*-tests were used to compare the imputation accuracy between NAMgbs and NAMskim for matching accessions.

### Wheat PHG database construction

The Wheat PHG database was built using PHG version 0.017. Instructions for creating the PHG along with source code are located with the PHG wiki: https://bitbucket.org/bucklerlab/practicalhaplotypegraph/wiki/Home. The approaches and parameters for constructing the Wheat PHG were discussed and developed during two PHG workshops organized at Cornell University. The first step of the PHG database construction is to create reference ranges for data storage and variant imputation (Supplementary Figure S1). In this case, “informative” reference ranges were chosen by extending the high confidence gene model coordinates from Chinese Spring RefSeq v.1.1 (IWGSC 2018) 500 bp in each direction. Adjacent ranges were merged if the boundaries lie within 500 bp from each other. This resulted in a final set of 106,484 informative reference ranges across the RefSeq v.1.1, while the remaining intergenic ranges were considered less informative due to abundance of repetitive sequences (Supplementary Figure S1).

The second PHG construction step populates the database with sequence data from diverse accessions across the reference ranges (Supplementary Figure S1). Pre-processed exome capture g.vcf files for the WC65 accessions, including 58 *T.* *aestivum* accessions, three *Aegilops tauschii* accessions, three *Triticum turgidum* subsp. *durum* wheat cultivars, and one *T.* *turgidum* subsp. *dicoccum* accession (Supplementary Table S2) generated by GATK (McKenna *et al.* 2010) were loaded into the PHG, creating a database of 6,705,472 haplotypes. This set of haplotypes should be representative of the haplotypic diversity in the wheat breeding programs within the United States.

The third PHG construction step creates consensus haplotypes for the reference ranges, using the diversity data from the WC65 accessions (Supplementary Figure S1). This step collapses the raw haplotypes into consensus haplotypes using a user-defined maximum divergence (mxDiv) parameter, which was set to 0.0001 for wheat. This parameter results in the clustering of raw haplotypes that contain <1 variant within 10,000 bp into a common haplotype. The value of the mxDiv parameter was based on prior diversity estimates in wheat (Akhunov *et al.* 2010; Jordan *et al.* 2015), and aimed at retaining a manageable number of haplotypes per reference range as described in Jensen *et al.* (2020). In addition to the mxDiv parameter, we set minTaxa = 1, which retains haplotypes present in only one accession and facilitates the imputation of rare haplotypes. Using these parameters, a total of 712,733 consensus haplotypes were detected, which is approximately 6.7 haplotypes per informative reference range, similar to ∼5 haplotypes per reference range reported in the sorghum PHG (Jensen *et al.* 2020).

### Imputation using the Wheat PHG

For imputation using PHG, low coverage sequence data (fastq) was aligned to the consensus haplotypes stored in the PHG database (Supplementary Figure S1) using minmap2 (Li 2018) program. A Hidden Markov model was used to infer the paths through the PHG that match the mapped reads while determining the missing haplotypes. The variants were imputed using the haplotype structure stored in the database, and exported as a vcf file. By using minReads = 0 parameter, variant calls were imputed for all variable positions in the Wheat PHG database. The resulting vcf file for the imputed genotypes were compared to high quality variant information for imputation accuracy as described above for each dataset.

### Phenotypic regression of imputed genotypes

We used a family of 75 RILs from the spring wheat NAM panel (Jordan *et al.* 2018), where both parents were included into the Wheat PHG database, to assess the effect of imputation on QTL mapping applications. We filtered the 1.457 million genotypes from PHG imputation of the GBS data generated for these 75 RILs to retain variants that segregate between the parental lines, and selected allele with frequencies ranging between 0.35 and 0.65 in the RIL population. These variants were subsequently thinned using PLINK (Purcell *et al.* 2007) to remove markers that had an *r*^2^ ≥ 0.6 within a 50 SNP window, stepping 10 SNPs at a time. The resulting set of 9806 markers with no missing data was used for stepwise regression (SR) mapping performed with the ICIM software v.4.1.0.0 (Meng *et al.* 2015) with markers entering and exiting the model with *P*-value < 0.0001. The estimates of the total number of CrossOvers (TCO) and the distal CrossOvers (dCO) were taken from the previous analyses of the spring wheat NAM population for family NAM1 (Jordan *et al.* 2018). Heading dates (HDs) were measured at three locations for two growing seasons (Montana, South Dakota, Washington) for the 75 RILs and three checks. Best linear unbiased predictions for each line were estimated using the following linear mixed model with *lmer* package in R:

HD=year+location+line+year (location)+line×year,

where location, year, and location nested within year are fixed variables, and the line and line-by-year interaction terms are random variables.

## Results

### The Wheat PHG database development

A Wheat PHG database was created using whole-exome capture data from a set of 65 wheat accessions, WC65 (Supplementary Table S2) contributed by the major US wheat breeding programs and the parental lines used for the genetic analyses of the yield component traits in WheatCAP (www.triticeaecap.org). This set of accessions was selected from a larger panel of nearly 250 wheat cultivars assembled in coordination with the US wheat breeding programs to build a genomic resource to be used as a reference panel for genotype imputation. This diverse set of 65 accessions is comprised of mostly spring and winter bread wheat cultivars, but it also included three accessions of the diploid ancestor of the wheat D genome, *A.* *tauschii* (accessions TA1615, TA1718, and TA1662/PI603230), and four accessions of tetraploid wheat (three *T.* *turgidum* subsp. *durum* wheat cultivars Langdon, Ben, and Mountrail and one domesticated emmer, *T.* *turgidum* subsp. *dicoccum*, accession PI41025).

For constructing the PHG, the wheat genome was split into a set of informative reference ranges that represent the high confidence gene models in the IWGSC RefSeq v.1.1 (IWGSC 2018). By using the predicted gene models to define reference ranges, we aimed to reduce the impact of erroneous genotype calling associated with the misalignments of sequence reads to the repetitive portion of the wheat genome (Wicker *et al.* 2018) on the estimation of LD and detecting haplotype blocks. A total of 106,484 reference ranges spanning all 21 chromosomes were defined (Supplementary Figure S1 and Table S3), with an average of 5,070 reference ranges per chromosome; chromosome 4D contains the lowest (3,612 ranges) and chromosome 2B harbors the highest (6,221 ranges) number of reference ranges.

**Figure 1:**
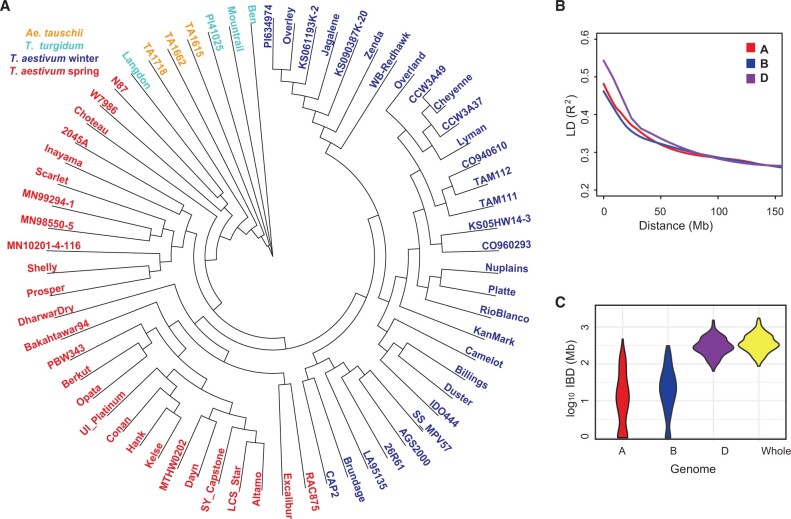
Genetic diversity of WC65 accessions of wheat and its diploid and tetraploid relatives used for developing the Wheat PHG. (A) Neighbor-joining tree of WC65 accessions used for constructing the Wheat PHG. (B) The rate of LD decay in the A, B, and D genomes of wheat. (C) The length of pair-wise IBD between the parental lines from different breeding programs used in WheatCAP.

Using the WC65 accessions to populate the Wheat PHG database, we discovered 1,473,670 SNPs and small-scale indels across the 106,484 reference ranges, of which 1,457,321 are high quality, bi-allelic SNPs (Supplementary Table S3). The inclusion of three diploid *A. tauschii* accessions into the panel increased the number of variable sites detected in the D genome lineage, which is the least polymorphic genome in bread wheat (Wang *et al.* 2013; Jordan *et al.* 2015; He *et al.* 2019). Excluding the variants from *A. tauschii*, we found that 161,226 (31%) sites in the D genome were monomorphic among the bread wheat cultivars. Similarly, we found that 31,486 SNPs (7%) in the A genome and 32,228 SNPs (6%) in the B genome are contributed by the domesticated emmer and durum lines, and are monomorphic in hexaploid wheat. These private SNPs explain the high levels of divergence between the domesticated emmer and *A. tauschii* accessions from the hexaploid wheat lines (Figure  1A). The patterns of genetic diversity and allele frequency distribution in the D genome compared to those in the A and B genomes were consistent with the known population bottleneck cased by polyploidization (Table  1): (1) diversity mean estimates for the D genome were <2.3-fold that of the A and B genomes (π_D_ = 0.076, π_A_ = 0.175, and π_B_ = 0.182; Table  1), (2) the estimates of Tajima’s D were lower in the D genome than in the A and B genomes (Tajima’s D_D_ = –2.19, Tajima’s D_A_ = –0.67, and Tajima’s D_B_ = –0.55, Table  1), (3) the mean MAFs were greater in the A and B genomes than in the D genome (MAF_A_ = 0.12, MAF_B_ = 0.12, and MAF_D_ = 0.05), and (4) LD drops to half of its initial value (*r*^2^ ≤ 0.33) at 20 Mb in the D genome, whereas in the A and B genomes LD drops to the same level at 12 and 10 Mb, respectively (Table  1, Figure  1B).

**Table 1:** Estimates of genetic diversity (π), minor allele frequency (MAF), Tajima’s D and linkage disequilibrium in the WC65 population used for constructing the Wheat PHG

Diversity statistic	A genome	B genome	D genome
No. SNPs	430,050	504,260	523,011
MAF	0.116	0.122	0.050
π (per bp)	0.175	0.182	0.076
Tajima’s D	–0.673	–0.552	–2.192
LD*^a^* (*r*^2^ ≤ 0.33)	12.2 Mb	9.8 Mb	20.0 Mb

a Distance at which LD drops to half of its initial value (*r*^2^ ≤ 0.33).

**Table 2:** Comparison of imputation accuracy between PHG and Beagle using exome capture data

DS75 accession	PHG 0.5×	PHG 0.1×	PHG 0.01×	Beagle 0.1×	Beagle 0.01×
Arthur	95.4%	93.8%	88.5%	90.4%	86.4%
Alice	96.7%	95.8%	91.5%	92.3%	88.9%
Antero	97.1%	96.4%	91.9%	93.6%	89.5%
Bess	96.0%	94.5%	89.2%	91.1%	86.6%
Branson	96.0%	94.4%	87.7%	91.3%	87.5%
Bolles	96.8%	95.4%	90.1%	88.6%	93.3%
BrawlCLPlus	96.3%	94.9%	91.3%	92.5%	88.6%
Byrd	96.8%	96.0%	92.7%	93.4%	88.9%
Camelot	98.0%	98.2%	97.5%	92.4%	88.0%
Danby	96.6%	95.8%	92.2%	93.4%	88.5%
Decade	96.3%	95.3%	91.1%	92.5%	88.7%
Denali	96.4%	95.5%	92.0%	92.2%	88.2%
DoubleCLPlus	96.9%	95.8%	90.6%	93.1%	89.0%
Duster^*a*^	97.7%	97.7%	97.1%	89.3%	93.0%
Expedition	97.0%	96.1%	92.7%	93.5%	89.0%
Forefront	96.3%	95.0%	89.6%	88.0%	91.7%
Freeman	96.4%	95.6%	91.4%	92.8%	87.5%
Glacier	96.4%	94.6%	88.2%	91.7%	87.4%
Gallagher	96.4%	95.2%	89.9%	91.3%	86.7%
Goodstreak	97.2%	96.0%	91.1%	93.7%	88.9%
Hilliard	95.9%	94.3%	89.0%	91.2%	86.9%
Hunter	95.2%	93.9%	87.8%	89.7%	85.7%
Hatcher	96.0%	95.4%	90.3%	92.4%	88.2%
Ideal	96.1%	95.7%	91.2%	91.6%	87.7%
Jamestown	96.1%	93.2%	89.7%	91.2%	86.0%
Jagger	95.9%	94.4%	90.6%	84.2%	75.6%
Jagalene	97.6%	98.0%	98.1%	93.0%	87.8%
Jerry	96.8%	95.8%	91.5%	93.3%	88.8%
KS061193K-2	97.5%	97.8%	97.9%	93.6%	88.5%
KS090387K-20	97.6%	97.9%	96.2%	92.1%	87.3%
KS13H-9	96.9%	96.0%	90.7%	93.1%	88.7%
KS14H-180-4	97.0%	96.2%	91.1%	93.0%	88.8%
KanMark	98.1%	98.2%	97.1%	93.3%	89.5%
Kharkof	96.2%	94.5%	90.4%	92.6%	88.6%
LCSChrome	96.3%	95.5%	90.1%	91.9%	86.9%
Linkert	97.0%	96.0%	91.5%	90.1%	93.8%
Lonerider	97.6%	95.9%	91.0%	92.6%	87.7%
Mace	96.7%	95.6%	90.2%	93.1%	88.7%
Mattern	96.6%	95.4%	91.9%	92.5%	87.9%
McGill	96.7%	95.6%	90.9%	93.0%	89.0%
Millenium	96.8%	95.8%	91.6%	92.8%	88.7%
Mott	96.4%	95.4%	90.4%	93.2%	89.6%
NE10589	96.8%	96.4%	91.9%	93.1%	88.1%
NUPlains^*a*^	97.9%	98.0%	96.7%	93.7%	89.7%
NW13493	96.6%	95.6%	90.7%	92.6%	87.4%
OK11D25056	96.8%	95.4%	91.2%	92.9%	88.9%
OK12716Red	96.5%	95.5%	90.9%	92.5%	87.4%
OK13209	96.9%	95.7%	91.0%	93.0%	88.7%
OK13621	96.9%	95.9%	91.5%	92.2%	87.3%
OK11709W-139122	96.7%	95.8%	91.9%	92.8%	89.2%
Oahe	96.4%	95.4%	91.1%	92.6%	88.9%
Overley*^a^*	97.2%	97.3%	97.2%	89.4%	92.9%
Pembroke	95.1%	93.3%	87.7%	89.4%	85.3%
Panhandle	96.2%	95.1%	90.4%	92.2%	87.4%
Prevail	96.5%	95.4%	89.8%	91.8%	89.7%
Redfield	96.5%	95.6%	90.8%	92.9%	88.5%
Robidoux	96.9%	95.9%	91.5%	93.2%	89.6%
SD08080	96.7%	95.7%	90.7%	92.7%	88.5%
Scout66	96.9%	95.9%	92.4%	93.7%	89.6%
Snowmass	96.6%	95.7%	91.0%	93.0%	88.3%
TAM114	96.7%	95.8%	92.0%	92.8%	89.3%
TAM203	96.1%	95.2%	91.1%	91.5%	86.9%
TAM204	95.8%	94.9%	90.9%	92.1%	87.7%
TAM303	96.0%	94.9%	91.6%	90.9%	87.1%
TAM304	96.7%	95.2%	90.1%	92.3%	88.6%
TAM305	96.4%	95.6%	90.9%	91.9%	87.1%
Traverse	96.7%	95.1%	90.3%	90.5%	86.6%
Tribute	95.6%	94.1%	87.0%	89.6%	85.0%
TX11A001295	96.9%	96.2%	93.8%	92.4%	87.4%
TX12M4068	96.5%	95.2%	91.6%	92.0%	87.4%
WB-Redhawk	97.7%	97.6%	98.1%	93.0%	88.6%
Wesley	97.0%	95.9%	91.9%	93.9%	89.9%
Yellowstone	95.8%	94.7%	91.1%	94.7%	93.2%
Zenda*^a^*	97.7%	97.7%	97.5%	93.1%	88.4%
Average	96.6%	95.7%	91.7%	92.1%	88.3%

a Cultivars used in PHG database construction.

**Table 3:** The accuracy of DS75 imputation in different wheat genomes

Wheat genome	**PHG (0.1**×**)^a^**	**Beagle (0.1**×**)^a^**	**PHG (0.01**×**)^a^**	**Beagle (0.01**×**)^a^**
Total	95.7%	92.1%	91.7%	88.3%
A	95.1%	91.2%	90.3%	85.4%
B	94.9%	90.4%	89.9%	85.5%
D	97.4%	96.6%	95.3%	94.6%

a Accuracies by approach are comprised of matching germplasm, EC: *n* = 75, Beagle: *n* = 75.

The accuracy and the rate of genotype imputation are affected by the proportion of shared genetic ancestry among individuals in a population (Browning and Browning 2013). For each WheatCAP parental line included in the Wheat PHG, we estimated the length of genomic segments sharing IBD with other lines in the panel. On average, the pairs of parents had 451 Mb (∼3%) of IBD segments (Supplementary Table S4), suggesting distant relationships among the WheatCAP parental lines. This result was consistent with the high correlation (*r *=* *0.64) observed between the genetic distance and IBD. However, the estimates of the total length of IBD segments among cultivars were quite variable (Figure  1C). For example, in cultivars Prosper from North Dakota and Shelly from Minnesota, the length of shared IBD segments was nearly 1.29 Gb (8.6%), whereas hard winter wheat cultivars Lyman (South Dakota) and Overley (Kansas) shared only 128 Mb (0.85%) of IBD segments. The average length of IBD segments shared by the distantly related durum wheat and domesticated emmer parents was only 57.6 Mb. Across all breeding programs, we detected 556 regions sharing IBD, with an average IBD segment length of 12.2 Mb. Over half (53%) of the IBD segments overlapped with a segment from at least one other breeding program, translating to more than 1.68 Gb of the genome shared between any two wheat breeding programs. This estimate includes 1.49 Gb of shared IBD in the D genome (89%), while only 86.4 Mb and 105.7 Mb of IBD with other breeding programs were detected in the A and B genomes, respectively. The genomic segments sharing IBD with most of the wheat lines were located on chromosomes 7D (568–571 Mb) and 3D (496.6–505 Mb), which were common to seven breeding programs.

The WC65 dataset included 21 hard red winter wheat cultivars from the US Great Plains region (Supplementary Table S2). Pairwise comparisons among these lines showed that, on average, they share 416 Mb of IBD segments, with an average IBD segment length of 13 Mb, and nearly 83% of all shared IBD regions are located in the D genome (Supplementary Table S5). This finding is consistent with the lack of diversity among breeding lines in the D genome (Chao *et al.* 2010) and the high levels of shared ancestry among the lines from the US Great Plains’ breeding programs.

### Genotype imputation using the Wheat PHG

We used several low-coverage sequencing datasets to assess the imputation performance of the Wheat PHG (Supplementary Table S2). First, we used a set of 75 spring and winter wheat lines, DS75, from the US wheat breeding programs sequenced using the whole-exome capture approach (Krasileva *et al.* 2017; He *et al.* 2019) to mimic a low-coverage sequencing experiment. We down-sampled the raw unmapped Illumina PE reads generated for each accession to create datasets with three levels of sequence coverage depths (0.01×, 0.1×, and 0.5×) for the regions targeted by the exome capture assay. The accuracy of imputation achieved using the Wheat PHG was estimated by comparing the concordance of imputed genotype calls with the genotype calls from the HQ-SNP set generated using the 90K iSelect array (Wang *et al.* 2014) and the high-coverage (20–30× coverage) exome sequencing.

**Figure 2:**
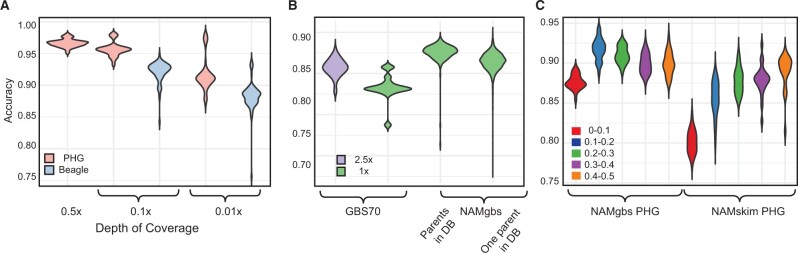
The accuracy of imputation using the Wheat PHG. (A) The impact of sequence coverage and the method of imputation on accuracy for DS75. (B) Accuracy of imputation using GBS sequencing at different coverage levels and different database haplotype representation. (C) Accuracy of imputation for alleles with different minor allele frequency for matched samples using GBS and skim-sequencing, *n* = 24.

On average, using 0.5× coverage of DS75, we achieved 96.6% imputation accuracy, ranging from 95% to 98% among lines (Figure  2A, Table  2). Five- and 50-fold reduction in the depth of read coverage for DS75 did not result in a substantial reduction in the accuracy of imputation. The mean accuracy of PHG imputation was 95.7% (93–98% range) with 0.1× coverage depth, and 91.7% (87–98% range) with as little as 0.01× coverage depth (Figure  2A, Table  2). These results suggest that the imputation method in the PHG could effectively use 0.01× exome coverage data to adequately capture the haplotypic diversity of the DS75 panel to achieve ∼92% imputation accuracy. The imputation accuracy of DS75 varied among the wheat genomes, likely due to genome-specific differences in the extent of LD and haplotypic diversity (Jordan *et al.* 2015). At 0.01× coverage depth, the accuracy of genotype imputation in the D genome was 95.3%, which was 5% and 5.4% more accurate [*P*-value_(ANOVA)_ < 2 × 10^−16^] than imputation in the A (90.3%) and the B genomes (89.9%), respectively (Table  3; Figure  2B).

We compared the performance of the Wheat PHG to one of the commonly used low-coverage imputation methods implemented in Beagle5.0 (Browning and Browning 2013). For this purpose, the WC65 panel of accessions included into the Wheat PHG database was used as the reference panel, and an independent set of DS75 wheat cultivars from the US wheat breeding programs was used as the inference panel. Overall, Beagle imputed missing genotypes with 88.3% accuracy for DS75 at 0.01× coverage (ranging from 76% to 94%), and 92.1% (ranging from 84% to 95%) at 0.1× coverage (Figure  2A, Table  2). Direct comparisons of imputation methods show PHG imputation statistically outperformed Beagle imputation by > 3.4% at both coverage levels [*P*-value _0.1__×__(__*t*__-test)_ = 2.0 × 10^−16^; *P*-value _0.01__×__(__*t*__-test)_ = 1.9 × 10^−14^).

Similar to the imputation of DS75 with PHG, Beagle imputed the D genome with higher accuracy [94.6%; *P*-value _(ANOVA)_ < 2 × 10^−16^] than both the A (85.4%) and B (85.5%) genomes (Table  3). The higher extent of LD in the D genome appears to contribute to more accurate genotype imputation compared to that in the A and B genomes using exome capture data, which show faster rates of LD decay and lower proportions of the genome sharing IBD segments in the panel used to build the PHG database.

We compared PHG imputation performance for four cultivars (Duster, Overley, NuPlains, and Zenda) in the DS75 panel that were included in PHG database construction, with respect to the other 71 accessions not included in the database construction, and found the four cultivar’s imputation accuracy was statistically higher (ANOVA for different levels of sequence coverage: *P*-value _0.5__×_ = 0.0008; *P*-value _0.1__×_ = 9.2 × 10^−5^; *P*-value _0.1__×_ = 3.8 × 10^−6^) than for other cultivars at all levels of sequence coverage (Supplementary Figure S2a). No similar relationship between the presence of specific haplotypes in the reference panel and imputation accuracy was observed for Beagle. We further explored this relationship by analyzing genotype imputation results in the cultivar Jagger, which showed a substantial reduction in imputation accuracy in the low sequence coverage datasets (0.1× and 0.01× coverage) imputed using Beagle (Supplementary Figure S2a). We assumed that one of the likely factors contributing to the decreased imputation performance of Beagle in the cultivar Jagger was the presence of the wild-relative introgression from *Aegilops* *ventricosa* on chromosome 2A (Cruz *et al.* 2016). Because cultivar Overley, which was used to build the PHG database, also carries this *A. ventricosa* introgression (Cruz *et al.* 2016), we could evaluate the impact of the presence of the rare introgressed haplotype in both the PHG database and the Beagle’s reference panel on imputation accuracy. The chromosome-by-chromosome assessment of imputation accuracy for cv. Jagger in the 0.01× coverage dataset showed modest accuracy (90%) for chromosome 2A using PHG. However, for the same chromosome, the imputation accuracy of Beagle reached only 63% (Supplementary Figure S2b). The accuracy of Beagle imputation was also low for other chromosomes (2D, 6A, 7A) (Supplementary Figure S2b), which suggests that cv. Jagger likely carries other regions with unique haplotypes (Kippes *et al.* 2018; Walkowiak *et al.* 2020) poorly represented in the reference set used for Beagle imputation. For the same three chromosomes, the accuracy of PHG imputation was higher than that obtained using Beagle.

#### Imputation accuracy with reduced coverage sequencing data

To this point, we tested the imputation accuracy using the same type of genomic data (whole-exome capture) as was used to populate the PHG database. We also evaluated the utility of the developed PHG database for imputing genotypes using two cost-effective complexity-reduced sequencing approaches, GBS (Elshire *et al.* 2011; Saintenac *et al.* 2013) and whole-genome skim-seq (Malmberg *et al.* 2018). We imputed a population of 70 independent accessions (GBS70) that were sequenced with GBS technology, to check imputation accuracy using sequencing reads derived from part of the genome that are not necessarily representative of the reference ranges in the database. Within the GBS70 accessions are 18 accessions that were sequenced at ∼1× the GBS target space and 52 sequenced 2.5× GBS target space. As anticipated, an increase in coverage increased imputation accuracy by 1.7% using GBS sequencing [Figure  2B, *P*-value _(ANOVA)_ < 4.2 × 10^−09^]. However, the imputation accuracy of 2.5× coverage GBS reads, which represents nearly 500× more sequencing reads per sample than DS75 at 0.01× was still reduced by 3.1% (Table  4), suggesting that matching sequencing reads derived from the reference ranges significantly increases imputation accuracy, even at substantially lower coverage depth.

**Table 4:** Comparison of imputation using complexity reduced sequencing technologies

Dataset	GBS70		NAMgbs		NAMskim
Coverage	1×	2.5×	1×	1×	0.1×
Avg. reads/sample	1.85 million	5 million	1.85 million	1.85 million	6.1 million*^a^*
Database status	Independent	Independent	Semi-dep.	Dependent	Semi-dep.
Imputation accuracy	86.9%	88.6%	89.2%	90.1%	85.3%

a Paired-end sequencing.

In addition to the 70 independent accessions characterized by GBS that were not used for PHG database construction, we utilized GBS reads generated for a set of 2,100 NAMgbs RILs from the spring wheat NAM panel (Jordan *et al.* 2018), and performed genotype imputation at 1.4 million variable sites. The common parent of these NAM RILs, cv. Berkut, was included into the Wheat PHG, and therefore this population does not necessarily represent an independent dataset for imputation as the GBS70 population did. However, for three families comprising the wheat NAM population, both parents were represented in the Wheat PHG, which allows us to investigate imputation accuracy for a set of RILs, which had either both or only a single parental haplotype being represented in the PHG database.

The mean accuracy of imputation across the 2100 RILs was 89.2%, ranging from 78% to 92% across individual lines (Figure  2B). Average imputation accuracies by families ranges from 88.3% to 90.4%, and the three families with both parents represented in the PHG database were among the top four most accurately imputed families (Supplementary Table S6). Even though there is only a 0.9% reduction [90.1% both parents; 89.2% single parent in database; *P*-value _(ANOVA)_ < 2 × 10^−16^] in mean imputation accuracy for lines with both parents in the database, *vs.* those with one parent, all lines having one or two parents represented in the database were imputed more accurately (3.2% and 2.3%, respectively) than the 18 independent lines from GBS70 with the same depth of coverage, whose accuracy was 86.9% (Table  4). These estimates of imputation accuracy for the semi-dependent (representation of parents in the PHG database) NAMgbs RILs were slightly lower (2.5%) than those observed for the imputed genotypes in the 0.01× DS75 exome capture data, and likely explained by the relatively small overlap (∼5%) between the sites in the GBS and exome capture datasets (Jordan *et al.* 2015). Overall, these results indicate that a PHG database created by a panel of independent wheat lines re-sequenced by exome capture assay provides accurate imputation (∼87%) on the inference populations created by complexity reduced sequencing using GBS, as long as the coverage is ∼1× GBS target size, and imputation is even more accurate for lines that share haplotypes represented in the PHG database.

We also evaluated the Wheat PHG imputation for a set of 24 NAM RILs genotyped using the whole-genome skim-seq approach (NAMskim). The genomic libraries generated for this set of RILs from the spring wheat NAM population (Jordan *et al.* 2018; Blake *et al.* 2019) were sequenced on an Illumina sequencer (2 × 150 bp run) to provide ∼0.1× genome coverage. The accuracy of PHG-imputed genotypes in the NAMskim dataset (85.3%) was lower than that obtained for genotypes in either the DS75 or 1x NAMgbs datasets (Table  4). In fact, this estimate was 3.9% lower for the same set of RILs [*P*-value _(__*t*__-test)_ < 2.7 × 10^−13^] imputed from the NAMgbs dataset. This lower accuracy likely is associated with a lower proportion of skim-seq reads, mostly represented by reads from the repetitive regions, uniquely mapped to the wheat genome compared to the proportion of uniquely mapped reads from the exome capture and GBS datasets, which are enriched for the low-copy genomic regions (Saintenac *et al.* 2013; Jordan *et al.* 2015). The accuracy of imputation varied across different SNP frequency classes. For SNPs with MAF > 0.1, the accuracy of imputation improved by 4% for all NAMgbs RILs, and by 7.5% for NAMskim genotypes (Table  5). The accuracy reached nearly 90% for NAMskim and 92.5% for NAMgbs datasets when the MAF were ≥0.2 (Table  5, Figure  2C).

**Table 5:** Relationship between minor allele frequency and the accuracy of imputation for reduced complexity semi-dependent datasets

	Minor allele frequency (MAF)					
	0–0.1	0.1–0.2	0.2–0.3	0.3–0.4	0.4–0.5	>0.1^*a*^
No. sites*^b^*	1,029,330	156,251	97,013	73,001	66,296	392,561
NAMgbs accuracy	0.8707	0.9226	0.9168	0.9078	0.9126	0.9134
NAMskim accuracy	0.8015	0.8560	0.8782	0.8789	0.8900	0.8760
Matched*^c^* NAMgbs Acc.	0.8763	0.9172	0.9102	0.8994	0.8992	0.9084

a Summary of all groups where MAF > 0.1.

b The sites within each MAF frequency bin were determined by frequency in the PHG database.

c Data from NAMgbs for the same 24 lines sequenced for NAMskim.

#### Genetic analyses of trait variation using the imputed genotypes

**Figure 3:**
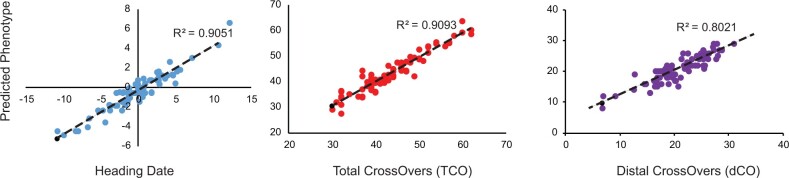
Relationship between the true and predicted phenotypes. Significant markers were identified by stepwise regression on heading date, total number of crossovers per line (TCO), and total number of distal crossovers per line (dCO) phenotypes.

The ability to accurately impute genotypes across the genome in low-coverage sequencing datasets provides a cost-effective means for advancing the genetic dissection of trait variation. We used the imputed PHG genotypes to assess the genetic contribution to HD variation in a NAM family previously used for studying the genetics of recombination rate variation in wheat (Jordan *et al.* 2018). The NAM1 family was chosen as both parents were included into the PHG database, and imputation accuracy was the highest among all NAM families at 90.4% (Supplementary Table S6). A SR was applied to identify variants associated with phenotypic variation. Before mapping, co-segregating redundant markers were removed, resulting in nearly 10,000 markers with no missing data. The SR method identified 11 SNPs together explaining 90% of the variance in HD, which was measured over 2 years at three locations (Figure  3, Supplementary Table S7). Among these SNPs are loci with modest effect sizes located on the long arms of chromosomes 5A and 5D, within 10 Mb from the *Vrn-A1* and *Vrn-D1* loci, which play a major role in the regulation of flowering in wheat (Distelfeld *et al.* 2009). In addition, significant SNPs on chromosomes 1B and 1D were mapped to the regions within 50 Mb of the *Elf-3* gene, which is associated with the transition from vegetative to reproductive growth in wheat (Alvarez *et al.* 2016; Zikhali *et al.* 2016).

We also used the imputed genotypes to revisit the genetic analysis of meiotic crossover rate variation in the wheat NAM population (Jordan *et al.* 2018; Blake *et al.* 2019). In the previous study, using a limited number of SNPs genotyped using the 90K iSelect array and GBS, we performed SR analysis and identified 15 and 12 SNPs associated with variation in the TCO and the number of dCO, respectively (Jordan *et al.* 2018). The identified SNPs explained 48.6% of the variation for TCO and 41% of the variation for dCO. Using the PHG imputed genotypes, we mapped 16 SNPs that together explained 91% of the variance for TCO per line and 12 SNPs explaining 80% of the variance for dCO (Figure  3, Supplementary Table S7). Compared to the previous study, SR analyses based on the PHG imputed SNPs detected additional loci with smaller effects on crossover rate (Jordan *et al.* 2018). As a result, the average effect size estimates for TCO and dCO were 2.5 COs and 1.5 COs, respectively. These estimates were lower than the previously reported average effect sizes of 3.36 COs for TCO and 2.3 COs for dCO (Jordan *et al.* 2018). Taken together, these results indicate that the increase in marker density after imputation using the Wheat PHG helped to identify new loci with a broader range of effect sizes that together explain a higher proportion of genetic variance compared to the previous study (Jordan *et al.* 2018).

## Discussion

We constructed a Wheat PHG database using wheat lines from the major US breeding programs and demonstrated that PHG combined with inexpensive low-coverage genome sequencing could be used to impute genotypes with high accuracy, sufficient to identify variants with smaller effects and support high-resolution mapping studies. Our analyses suggest that the Wheat PHG has the potential to effectively utilize community-generated whole-exome capture datasets, currently including thousands of diverse wheat accessions from different geographic regions (Molero *et al.* 2018; He *et al.* 2019; Pont *et al.* 2019; Scott *et al.* 2021), to create a global resource for imputing genotypes. The imputation accuracy provided by the PHG in populations genotyped using skim-seq, GBS, as well as low-coverage exome sequencing approaches varied, but overall were comparable, indicating that the marker density in the large populations of wheat lines previously genotyped using these methods could be substantially increased by imputation with this newly developed Wheat PHG tool. In addition to improved imputation accuracy, another attractive feature of the Wheat PHG for imputation is its ability to directly use sequence data in the fastq format, which significantly simplifies and reduces time required for data processing.

The accuracy of PHG imputation compared favorably with the commonly used imputation tool Beagle v.5.0 (Browning and Browning 2013), which imputed genotypes with 3.3% and 3.6% lower accuracy at 0.01× and 0.1× genome coverage levels, respectively. The Wheat PHG showed a substantial improvement in accuracy (10–15%) compared to Beagle for the cultivar Jagger that carries introgression from a wild relative that was represented in only one accession in the PHG database, indicating that PHG is more effective at utilizing the rare haplotypes in the reference panel than Beagle. In previous studies, imputation of exome capture data with Beagle in populations genotyped using the 90K SNP array and GBS was 93–97% (Jordan *et al.* 2015) and 98% (Nyine *et al.* 2019), respectively. These estimates of accuracy are slightly higher than those obtained in our current study, but overall are comparable, and likely associated with filtering applied to reduce the proportion of missing data in the imputed datasets (Nyine *et al.* 2019), and with the inclusion of more common variants from the array-based genotyping methods.

Compared to the imputation accuracy of sorghum (94.1%) and maize (92–95%) PHGs (Jensen *et al.* 2020; Valdes Franco *et al.* 2020), our estimates of accuracy were slightly lower and are likely caused by genotyping errors associated with the misalignment of short reads to the more complex, highly repetitive, allopolyploid wheat genome. The higher imputation accuracy in the low-coverage DS75 datasets from the whole exome capture compared to the accuracy of whole genome skim-seq datasets, which are mostly composed of reads from the repetitive regions of the wheat genome, supports this explanation.

Our results show a reduction in the accuracy of imputation in the regions preferentially located outside of the reference ranges, for example in the regions around the PstI sites sequenced by GBS. We show that imputation accuracy within the reference ranges with lower depth of coverage, for example in the DS75 dataset providing at 0.01× coverage of the exome capture regions, is higher (92%) compared to PstI sites with higher sequence coverage, ∼1× in the GBS database (89%), even for accessions that are included into the PHG database. One possible approach to improve imputation accuracy for GBS datasets could be to create reference ranges around the GBS-associated PstI sites. However, this may also increase the proportion of ranges located within the repetitive portion of the wheat genome and increase the chance of read misalignment, reducing imputation accuracy.

The imputation accuracy among different allele frequency classes improves with an increase in the allele frequency and is higher for a reference allele than for an alternative allele. Consistent with these expectations, the accuracy of imputation in the GBS dataset improved from 87.1% for SNPs with MAF < 0.1 to 91.3% for SNPs with MAF > 0.4, and in the skim-seq dataset from 80.2% for SNPs with MAF < 0.1 to 89.0% for SNPs with MAF >0.4. Previous studies showed that an increase in the reference population size also increases the probability of capturing rare alleles and substantially improves the imputation accuracy of rare variants (Shi *et al.* 2017; Das *et al.* 2018). Our results suggest that the Wheat PHG appears to be more effective at utilizing rare haplotypes included into the reference panel for genotype imputation than the commonly used low-coverage imputation method from Beagle. This was demonstrated by imputing genotypes on chromosome 2A, which carries an introgression from *A. ventricosa* in cultivar Jagger (Cruz *et al.* 2016). The inclusion of genotyping data from the cultivar Overley, which also carries this *A. ventricosa* introgression, into the PHG database was sufficient for accurate imputation in Jagger. In spite of including genotyping data from cultivar Overley into the reference panel, Beagle imputation of chromosome 2A genotypes in Jagger was lower compared to PHG. Further efforts aimed at broadening the diversity of accessions in the Wheat PHG, including wheat lines carrying known introgressions from wild relatives, will be needed to improve the utility PHG tool for genotype imputation in wheat germplasm.

The application of imputed genotypes to the genetic analyses of trait variation in the wheat NAM population showed that an increase in marker density increases the number of loci associated with trait variation and detects alleles that have smaller effects on phenotypes (*e.g.* recombination rate) than those previously detected using lower density marker sets. The increase in the number of significant loci also resulted in a higher proportion of genetic variance (80–91%) in recombination rate and HD being explained, suggesting that the imputed genotypes are better at capturing the genetic architecture of these traits, and have the potential to identify more adaptive and beneficial genetic targets in breeding programs.

## Data availability

The raw sequence data for previously published accessions can be accessed from the NCBI Short-Read Archive database (BioProject SUB2540330 and PRJNA381058). Newly generated exome capture data can be accessed from NCBI Short-Read Archive database (BioProject PRJNA732645). Genotypic datasets used in this study are available from the website: http://wheatgenomics.plantpath.ksu.edu/phg/ (Accessed: 2021 November 23) Phenotypic datasets for NAM Family 1 associated with the paper can be downloaded from the wheat NAM project website: http://wheatgenomics.plantpath.ksu.edu/nam/ (Accessed: 2021 November 23). Supplemental Material available at figshare: https://doi.org/10.25387/g3.14770974.
